# Application of vigor indexes to evaluate the cold tolerance in rice seeds germination conditioned in plant extract

**DOI:** 10.1038/s41598-021-90487-x

**Published:** 2021-05-26

**Authors:** Sheila Bigolin Teixeira, Stefânia Nunes Pires, Gabriele Espinel Ávila, Bruna Evelyn Paschoal Silva, Victoria Novo Schmitz, Cristiane Deuner, Rodrigo da Silva Armesto, Diogo da Silva Moura, Sidnei Deuner

**Affiliations:** 1 Postgraduate Program in Seed Science and Technology, Eliseu Maciel Faculty of Agronomy, Federal University of Pelotas, Pelotas, Rio Grande do Sul 96010-900 Brazil; 2 Postgraduate Program in Plant Physiology, Department of Botany, Institute of Biology, Federal University of Pelotas, Pelotas, Rio Grande do Sul 96010-900 Brazil; 3 Eliseu Maciel Faculty of Agronomy, Federal University of Pelotas, Pelotas, Rio Grande do Sul 96010-900 Brazil; 4 Riograndense Higher Education Center, Marau, RS 99150-000 Brazil

**Keywords:** Developmental biology, Plant sciences, Environmental sciences

## Abstract

Rice is a crop that presents sensitivity to cold, especially in the germination phase, which leads to high economic losses. Alternative management forms are essential to increase tolerance to low temperatures, and seed priming represents a promising tool. The objective of this study was to investigate the priming effect of the aqueous extract of carrot roots on rice seeds to increase tolerance to low temperatures during germination. Seeds from cultivars BRS Querência (cold-susceptible) and Brilhante (cold-tolerant) were soaked for 24 h in concentrations of 0, 25, 50, and 100% carrot extract, sown on germitest paper and conditioned in BOD for 21 days at 15 °C. As a control, the seeds soaked in water were also germinated at 25 °C. They were evaluated for germination, first germination count, and germination speed index to calculate the stress indices: tolerance index, susceptibility index, and harmonic mean. They were also evaluated for the length and dry mass of shoot and root. The results showed that the rice seeds conditioning in carrot extract effectively reduces the damage caused by cold, significantly increasing the germination speed and the percentage of final germination and the growth evaluations, more expressive at 100% concentration. The stress indexes are efficient in estimating the tolerance of the cultivars and the effect of the different conditions in low-temperature conditions, highlighting the superiority of the Brilhante cultivar.

## Introduction

Plants can suffer limitations in development and growth due to environmental changes, including extreme events such as droughts, floods, high or low temperatures. Such adverse conditions cannot be controlled, so crop losses are considered inevitable. Studies suggest that more than 60% of the fluctuations in the annual yield of essential crops are attributed to climatic variability^1,2^.

Food security and biodiversity conservation are essential issues discussed to assess decisions to increase agricultural production through sustainable intensification^3^. Studies indicate that the world population will exceed nine billion by 2050^4^, and from this scenario, it is necessary to increase food production, quantity, and quality, to meet the nutritional needs of the world population.

Rice is one of the essential foods since it is grown in more than 100 countries and often consumed by more than 3.5 billion people worldwide, a number that represents almost half of the world population^5^. This culture can be grown in different environments depending upon water availability and temperature conditions. However, low temperature is a significant factor limiting rice growth and yield, and the seedling is one of the developmental stages at which sensitivity to cold stress is higher, being a recurrent problem in several regions of the world^6,7^, including Brazil, mainly in the southern area of Rio Grande do Sul (RS), state responsible for more than 70% of national production^8^. Still, globally, more than 15 million ha of rice is damaged by the low temperature at the seedling and reproductive stages. Because of low temperature, nearly 7 million ha of potential rice land in South and Southeast Asia remain unplanted, dominated by cultivars of the indica subspecies that possess tropical origin being very sensitive to low temperature^9^.

As reported by^10^, plants are classified by the intrinsic tolerance level to low temperatures. Rice cultivars of the subspecies japonica present more tolerance to cold than those of the subspecies indica, due to their origin in places with different climatic characteristics^7,11^, being the cold tolerance the immediate solution to damage. The great majority of rice cultivars used to come from tropical origin, so the optimum temperature of cultivation is between 20 and 35° C, varying accordingly to the stage of crop development. For this reason, temperatures below 20 °C are a limiting factor in a large part of the crop cycle, mainly in the germination, seedling development, and reproductive stages were depending on the stress duration, the damage can be very severe for the culture^12,13^.

In the State of Rio Grande do Sul (RS), low winter temperatures often extend into the spring during September and October, timing in which many producers carry out sowing so that the reproductive stage coincides with the highest solar radiation months, December and mainly January. Consequently, seed germination and seedling's emergence can be delayed by more than 20 days, especially in the most sensitive cultivars. The irrigation water temperature is another factor that affects the irrigated rice from the initial irrigation phase until the beginning of the panicle formation. The crop is more affected by the water temperature than the air^14^.

In order to obtain a higher percentage of germination and plant establishment speed, particularly under adverse climatic conditions, has been used the technique of seed priming^15–17^. This technique has been well studied, mainly using water or solutions with known osmotic potentials. However, there is little study related to using a substance that aggregates desired characteristics to seed, such as plant extracts. Many plants are rich in bioactive compounds with high antioxidant potential, which minimizes the effects of stress on plants by preventing oxidative damage caused by reactive oxygen species^18,19^. Among the plants with high antioxidant capacity, carrots (*Daucus carota* L.) stand out, mainly due to the significant amount of carotenoids and phenolic compounds and the presence of tocopherols, nitrogen compounds, and vitamins, among others. These facts seem to suggest that extracts from many different kinds of plant materials contain typical growth-promoting substances, which may be involved in the mechanism of induction of growth in various kinds of plant tissues^20,21^.

Therefore, the objective of this study was to investigate the priming effect of the aqueous extract of carrot roots on rice seeds in response to tolerance to low temperatures during germination. Vigor indexes were analyzed in order to obtain this data.

## Material and methods

The experiment was conducted in a completely randomized design, in a factorial arrangement with four replications; factor A included two rice cultivars: BRS Querência (ssp. Indica: cold-susceptible) and Brilhante (ssp. Japonica: cold-tolerant) and factor B, included four treatments for seed conditioning: 0, 25, 50, and 100% aqueous carrot extract. All methods were carried out in accordance with relevant guidelines and regulations.

Seeds of the rice were disinfected with sodium hypochlorite (2% active chlorine) for 5 min and then placed in beakers containing solutions of 25%, 50%, and 100% aqueous carrot extract, and kept under soaking for 24 h at 15 °C. Two hundred and fifty grams of carrot roots of the cultivar Kuronan, which has cylindrical roots with a uniform dark-orange coloration, were processed in a centrifuge (model Mondial Premium 700 W) at room temperature, obtaining a volume of 100 mL of crude extract. The extract was prepared on the day of its use, and the concentration of carrot extract was diluted with ultra-pure water (v/v). The resulting extract was diluted in ultra-pure water to obtain the different established percentages, being characterized by pH, osmotic potential (Ѱs), electrical conductivity (EC), and β-carotene content (Table 1).

**Table 1:** Characterization of the carrot extract as pH, osmotic potential (Ѱs), electrical conductivity (EC), and β-Carotene content.

Carrot extract (%)	pH	Ѱs (MPa)	EC (mS cm^−1^)	β-Carotene (mg mL^−1^)
25	6.04	− 0.13	1.19	0.0109
50	6.04	− 0.31	1.98	0.0171
100	6.04	− 0.61	3.63	0.0352

After soaking, the seeds were sown on “germitest” paper moistened with water to a volume of 2.5 times their weight and were kept in a germinating chamber regulated at 15 °C with a photoperiod of 12 h. As a control, seeds were soaked in distilled water (0%), germinated at 15 °C and 25 °C, forming the negative and positive control, respectively.

The germination test was conducted with 200 seeds (four subsamples of 50 seeds), as specified by the Rules for Seed Analysis^22^. The first germination count (FGC) was carried out together with the germination test and evaluated on the tenth day after sowing. The germination speed index (GSI) was also carried out simultaneously with the germination test by counting the number of normal seedlings with seeds with a root extension equal to or greater than 2 mm until the number of normal seedlings remained constant^23^. However, the root and shoot length (RL and SL) were determined after 21 days from the beginning of the test because a low temperature was applied (15 °C), and the development of rice at low temperatures occurs more slowly than in suitable temperature such as 25 ºC. Thus, RL and SL were determined in 10 seedlings of each sub-sample, randomly assessed for each treatment using a graduated ruler. These seedlings were then separated and transferred to an oven at 70  ± 2 °C until a constant mass was obtained to determine the dry mass of the shoots and roots.

The seedling vigor index was calculated according to the equation^24^, where: SVI = (SL + RL) × G %. With the data obtained from GSI, the following indices were also calculated: Cold stress tolerance index (STI = (Yp × Ys)/Xp^2^); cold stress susceptibility index (SSI = (1 − (Ys/Yp))/(1 − (Xs/Xp))), proposed by^25^ and harmonic mean, (HM = 2 × (Yp × Ys))/((Yp + Ys))), according to^26^, where: Ys: corresponds to GSI of each treatment with stress environment (15 °C); Yp: corresponds to GSI of cultivar in the non-stress environment (25 °C); Xs: corresponds to the mean of the GSI of the control of both cultivars in a stress environment (15 °C); Xp: corresponds to the mean of the GSI of the control of both cultivars in the non-stress environment (25 °C).

Data were subjected to analysis of variance (P ≤ 0.05), and the means were compared by Tukey’s test, at 5% probability. When a significant effect was found, these were tested by polynomial regression models using the SigmaPlot program, version 12.5, and the means compared using the R software^27^.

## Results

Data analysis showed a significant interaction between cultivars and seed conditioning treatments. The low temperature (15 °C) resulted in a significant reduction in the first germination count (FGC), the percentage of germinated seeds (G), and the germination speed index (GSI) in both cultivars however, cv. Brilhante was superior to BRS Querência in all parameters evaluated (Table 2).

**Table 2:** First germination count (FGC), germination test (G), and germination speed index (GSI) of rice seeds, cv. BRS Querência and cv. Brilhante in response to conditioning on carrot extract and low temperature.

	Cultivars	25 °C	25 °C			
		Control	Carrot extract (%)			
			0	25	50	100
FGC (%)	BRS Querência	80 ± 1.4 Ba	17 ± 2.8 Bd	31 ± 6.0 Bbc	24 ± 2.0 Bcd	34 ± 4.1 Bb
	Brilhante	89 ± 3.4 Aa	23 ± 3.0 Ac	72 ± 6.6 Ab	75 ± 3.0 Ab	77 ± 1.4 Ab
	CV (%)	7.8				
G (%)	BRS Querência	90 ± 6.3 Aa	37 ± 8.0 Bc	67 ± 6.4 Bb	72 ± 4.6 Bb	69 ± 3.8 Bb
	Brilhante	92 ± 3.0 Aa	64 ± 4.7 Ab	88 ± 4.3 Aa	89 ± 3.0 Aa	91 ± 4.8 Aa
	CV (%)	6.1				
GSI	BRS Querência	34 ± 0.7 Ba	3.3 ± 0.17 Bc	6.1 ± 0.2 Bb	6.2 ± 0.4 Bb	6.3 ± 0.6 Bb
	Brilhante	54 ± 1.9 Aa	5.4 ± 0.45 Ac	10.1 ± 0.6 Ab	10.0 ± 0.4 Ab	11 ± 0.6 Ab
	CV (%)	6.0				

Means followed by the same letter, upper case in the column and lower case in the row, do not differ by Tukey's test (P ≤ 0.05).

*CV* coefficient of variation, * ± SD* standard deviation.

Comparing the germination test at 25 °C with 15 °C for the seeds conditioned in water (positive and negative control), there was a reduction of more than 70% in FGC and 90% in GSI, for both cultivars, showing the negative effect of low temperature on the speed of germination, even in cv. Brilhante, considered tolerant. In the germination test, cv. Brilhante suffered a reduction of only 30.6%, compared to 59.4% for cv. BRS Querência.

However, analyzing the effect of conditioning treatments on rice seeds, carrot extract significantly responded to the evaluated parameters, resulting in the maintenance of seed vigor under low temperature in germination (Table 2). Although the tests of FGC, G, and GSI for conditioning in carrot extract, in general, had shown superiority to that observed in water conditioning, when germination at 15 °C, the best response was observed for treating 100% of the extract, reaching a percentage of 91% of germinated seeds for cv. Brilhante, very close to the 91.5% observed in seeds germinated at 25 °C, sets the optimum temperature for germination of rice.

**Figure 1:**
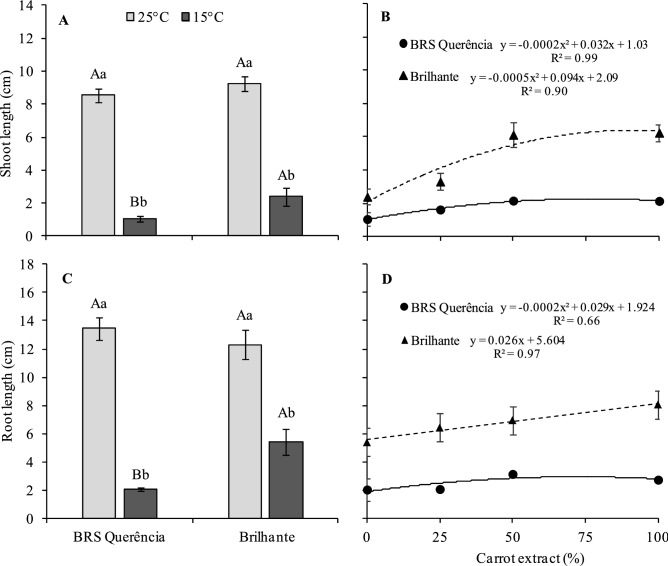
Shoot length of controls **(A)**, shoot length of treatments germinated at 15 °C **(B)**, root length of controls **(C)**, and root length of treatments germinated at 15 °C **(D)** of rice seedlings of cultivars BRS Querência (filled circle) and Brilhante (filled triangle). Means followed by the same letter, upper case in the comparison of the cultivars and lower case in temperatures, do not differ by Tukey's test (P ≤ 0.05). Bars represent the standard error of the mean of four replicates.

The effect of temperature variation on rice can also be observed in the evaluation of seedling growth parameters. When germinated at 25 °C, shoot length (SL) and root length (RL) showed no significant difference between the two cultivars studied (Fig. 1A,C). However, under 15 °C, there was a significant reduction for these variables, more expressive in cv. BRS Querência. In response to carrot extract, the SL increased with the extract on germination at 15 °C, reaching the maximum point at 80% and 94% of extract for the cv. BRS Querência and the cv. Brilhante, respectively (Fig. 1B). For the RL, the cv. Brilhante linearly increased with the use of the extract, while the cv. BRS Querência reached the maximum point with 72% extract (Fig. 1D).

**Figure 2:**
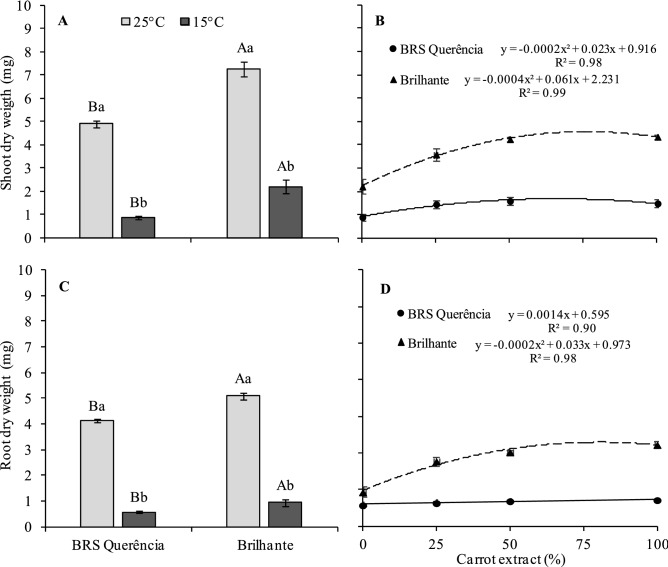
Shoot dry mass of controls **(A)**, shoot dry mass of treatments germinated at 15 °C **(B)**, root dry mass of controls **(C)** and root dry mass of treatments germinated at 15 °C **(D)** of rice seedlings of cultivars BRS Querência (filled circle) and Brilhante (filled triangle). Means followed by the same letter, upper case in the comparison of the cultivars and lower case in temperatures, do not differ by Tukey's test (P ≤ 0.05). Bars represent the standard error of the mean of four replicates.

The shoot dry mass (SDM) and root dry mass (RDM) showed a significant difference between the germination treatments at 25 °C, 15 °C and between the cultivars for the same germination condition (Fig. 2A,C). At 15 °C was observed less dry mass of the seedlings, but seedlings from conditioned seeds in carrot extract showed an increase in SDM, reaching the maximum point with 58% and 77% of extract in BRS Querência and Brilhante, respectively (Fig. 2B). For RDM, cv. BRS Querência presented positive linear behavior with the extract use, but it was not very expressive, increasing only 0.1 mg of the treatment with 0% to 100% of the extract and the cv. Brilhante reached the maximum point with 82% of extract (Fig. 2D).

The tolerance indexes (STI) were used to determine the level of tolerance to each cultivar's cold stress and test how much the carrot extract can increase this factor. The higher the index (closer to 1) and the harmonic mean (HM) (closer to the non-stress GSI), implies that the treatment is closer to the maximum response of the cultivar (control at 25 °C), and the opposite occurs with the stress susceptibility index (SSI).

Based on the calculations of STI, SSI, and HM, it was observed that cv. Brilhante is more tolerant to cold stress than cv. BRS Querência (Table 3) and the use of conditioning in carrot extract improve the cold tolerance in germination, as it increases the STI, together with the HM, and decreases the SSI. The SVI also showed a similar result, indicating the cv. Brilhante, with greater vigor and being an index that considers both the germination and the total length of the seedlings, can differentiate better the extracts treatments, standing out the treatment with 50% of extract for the cv. BRS Querência and 100% for cv. Brilhante. The conditioning with carrot extract presented more significant results in the susceptible cultivar (BRS Querência) since the indexes evaluated showed a higher percentage of increase in this cultivar.

**Table 3:** Stress tolerance index (STI), stress susceptibility index (SSI), harmonic mean (HM), and seedling vigor index (SVI) of two rice cultivars, BRS Querência and Brilhante, in response to the conditioning of the seeds in the extract of carrot and the low germination temperature.

Carrot extract (%)	BRS Querência				Brilhante			
	STI	SSI	HM	SVI	STI	SSI	HM	SVI
0	0.06 ± 0.003 b	0.89 ± 0.01 a	6.1 ± 0.300 b	39.6 ± 8.08 c	0.15 ± 0.01 c	0.89 ± 0.01 a	9.8 ± 0.740 c	71.3 ± 4.97 c
25	0.11 ± 0.003 a	0.80 ± 0.01 b	10.3 ± 0.25 a	70.2 ± 3.88 b	0.28 ± 0.02 b	0.79 ± 0.01 b	17.0 ± 0.96 ab	97.8 ± 3.52 b
50	0.11 ± 0.010 a	0.80 ± 0.02 b	10.5 ± 0.68 a	77.3 ± 4.46 a	0.28 ± 0.01 b	0.79 ± 0.01 b	16.8 ± 0.58 b	101.6 ± 3.57 ab
100	0.11 ± 0.010 a	0.80 ± 0.02 b	10.6 ± 0.85 a	73.9 ± 4.09 ab	0.30 ± 0.02 a	0.77 ± 0.01 b	18.2 ± 0.94 a	105.3 ± 3.84 a

Means followed by the same letter in the column do not differ from each other by the Tukey’s test (P ≤ 0.05).

*± SD* standard deviation.

With the data of Pearson linear correlation was possible to verify that the STI, SSI, HM, and SVI had a high correlation with all data measured, not only viability but also growth parameters (Table 4). Only the root dry mass did not show a high correlation for cv. BRS Querência, but was significant by t test. The HM, despite the high correlation, was not significant with the extract, in both cultivars, by t-test.

**Table 4:** Pearson correlation for 12 variables, evaluated in the germination season in two rice cultivars, BRS Querência (above diagonal) and Brilhante (below diagonal), in response to the conditioning of the seeds in carrot extract and the low temperature in germination.

	Extract	GSI	FGC	G	SL	RL	SDM	RDM	STI	SSI	HM	SVI
Extract	1	0.70**	0.65**	0.67**	0.74**	0.60*	0.58*	0.45^ns^	0.70**	− 0.71**	0.70**	0.68**
GSI	0.77**	1	0.79**	0.96**	0.75**	0.54*	0.83**	0.49^ns^	0.99**	− 1.00**	1.00**	0.96**
FGC	0.73**	0.98**	1	0.70**	0.42^ns^	0.17^ns^	0.60*	0.54*	0.78**	− 0.78**	0.79**	0.69**
G	0.71**	0.97**	0.95**	1	0.77**	0.61*	0.84**	0.44^ns^	0.96**	− 0.96**	0.97**	1.00**
SL	0.76**	0.58*	0.63**	0.53*	1	0.76**	0.75**	0.51*	0.74**	− 0.76**	0.76**	0.79**
RL	0.72**	0.66**	0.62**	0.63**	0.46^ns^	1	0.66**	0.52*	0.52*	− 0.55*	0.54*	0.64**
SDM	0.81**	0.89**	0.91**	0.88**	0.81**	0.61*	1	0.71**	0.82**	− 0.84**	0.83**	0.85**
RDM	0.83**	0.93**	0.91**	0.89**	0.73**	0.61*	0.93**	1	0.46^ns^	− 0.49^ns^	0.48^ns^	0.44^ns^
STI	0.76**	1.00**	0.98**	0.97**	0.58*	0.66**	0.89**	0.92**	1	− 0.99**	0.99**	0.96**
SSI	− 0.77**	− 1.00**	− 0.98**	− 0.97**	− 0.60*	− 0.65**	− 0.89**	− 0.93**	− 1.00**	1	− 1.00**	− 0.96**
HM	0.76**	1.00**	0.98**	0.97**	0.58*	0.65**	0.89**	0.93**	1.00**	− 1.00**	1	0.96**
SVI	0.79**	0.97**	0.96**	0.99**	0.64**	0.70**	0.93**	0.92**	0.97**	− 0.97**	0.98**	1

** Significant at 1% probability of error; * significant at 5% probability of error; ^ns^ not significant.

## Discussion

Seed germination and seedling emergence are the initial steps for establishing a culture, influenced by several environmental factors, such as temperature, water, light, and seed burial depth^28,29^. Temperature is one of the primary factors affecting the percentage and speed of germination, which directly works via seed imbibition and the biochemical reactions that regulate the metabolism involved in the germination process^30^.

Thus, the low-temperature stress at the seedling stage is a major constraint to rice production^13^. As can be observed in this study, there were sharp reductions in the germination and growth of the rice seedlings of the cultivars BRS Querência and Brilhante when subjected to low temperature, 15 °C (Table 2 and Fig. 1). Similar results were observed by^12,13,31^, studying rice genotypes in which the cold negatively affected germination, root length, and coleoptile length. According to^32^, the temperature has a significant effect on the length of the coleoptile and radicle, on the percentage of damage to the seed and germination rate in a germination period under stress conditions. Thus, characteristics such as germination rate, the root, and coleoptile length have been used in cold tolerance studies on germination^11,33^.

Also reported by other authors^34^, low temperature at the seedling stage may result in addition to poor germination in stunted seedlings, yellowing or withering, and reduced tillering in rice. With this study, it was possible to verify that the highest losses were observed for cv. BRS Querência, probably related to its origin.

The adverse effects of several abiotic stress factors are often mitigated by the application of natural and artificial growth promoters, or compounds with antioxidant activity^16,35^, which can be applied in different ways, such as spraying or priming of seeds by presoaking in these compounds. Seed priming is a cheap, quick, and straightforward methodology accessible to farmers in order to achieve higher germination rates and may be taken to counteract the adverse effects of abiotic stresses^17,36–40^.

According to^38^, carrot root extract can be used to stimulate plant growth because it is known to contain a variety of plant growth-stimulating compounds. Still, according to these authors, the analysis of carrot roots by HPLC showed that its extract includes a high content of vitamin A as β-carotene, protein, carbohydrates, fat, and vitamins B1, B2, B6, C, D, and E with notable antioxidant that reduce free radicals and cell injury.

In the present study, the positive effect of rice seed conditioning on carrot extract on germination and vigor tests in response to low temperature can be observed (Table 2). The results show that the best responses were obtained when the seeds were soaked (seed priming) in the pure extract (100%), where the cv. Brilhante maintains a percentage of germination equal to the control (germination at 25 ºC). This behavior may be associated with the carrot extract's B-carotene content (0.0352 mg mL^−1^) (Table 1). Several studies have reported the effect of the use of aqueous extracts on seed germination in response to saline stress^36,41,42^, drought stress^38^, and cold^43^.

The growth parameters evaluated also positively affected the extract, which practically doubled with 100% extract compared with the negative control, mainly observed in cv. Brilhante (Figs. 1 and 2). The tolerance of plants to cold stress depends mainly on the ability of their membranes to prevent injury and maintain their integrity^44^. In this sense, studies report that carrot root extract can reduce cell damage caused by free radicals, protecting the cell membranes mainly due to the presence of antioxidant compounds^38,45^.

The results of the stress tolerance indexes provided information that allowed to select the most tolerant to cold, but they were not efficient in the selection of the best percentage of carrot extract to increase the rice tolerance under cold stress, showing that these indexes do not work with minor differences in vigor (Table 3). Other studies have also found superior performance of the cultivar Brilhante, with greater growth in relation to the other cultivars tested^10,46^. However, the use of seedling vigor index provided sufficient data for the selection of the best treatment of carrot extract for each cultivar, as well as an efficient selection of the best kelp extract in the growth of tomato seedlings^47^, because it is a particular index, taking into account both germination and seedling length, variables which are very responsive to cold stress and conditioning in the extract.

## Conclusion

Carrot roots extract mitigates the cold effects on germination and initial growth of rice plants, both sensitive (BRS Querência) and tolerant (Brilhante) cultivars. The germination followed by growth evaluations provided sufficient information for the differentiation of the cultivars and the treatments with carrot extract, concerning the greater cold tolerance, through the indexes of tolerance to stress and vigor of seedlings.
